# Stroke in Young Adults: An Overview and Non-Pharmacological Preventive Strategies

**DOI:** 10.3390/brainsci15040375

**Published:** 2025-04-03

**Authors:** Aleksandar Sič, Nikola Andrejić, Jovana Ivanović, Vidna Karadžić Ristanović, Selena Gajić, Danka Bjelić, Marko Baralić, Nikola Stojanovic

**Affiliations:** 1 Faculty of Medicine, University of Belgrade, Dr. Subotića Starijeg Str. 8, 11000 Belgrade, Serbia; anikola99@yahoo.com (N.A.); marko.baralic@med.bg.ac.rs (M.B.); 2 Neurology Clinic, University Clinical Centre of Serbia, Dr. Subotića Starijeg Str. 6, 11000 Belgrade, Serbia; jo_ivanovic@yahoo.com; 3 Department of Nephrology, University Clinical Centre of Serbia, Pasterova Str. 2, 11000 Belgrade, Serbia; vidnakaradzic@gmail.com (V.K.R.); selenagajic@yahoo.com (S.G.); dankamedicus@gmail.com (D.B.); 4 Department of Physiology, Faculty of Medicine, University of Niš, Bulevar Dr. Zorana Đinđića Str. 81, 18000 Niš, Serbia

**Keywords:** stroke, young adults, prevention, ischemic stroke, hemorrhagic stroke, smoking cessation, mental health, public health

## Abstract

Stroke is one of the most common causes of death and disability worldwide, with significant impact on both physical and cognitive health. Although strokes are less common in young adults, they still occur in this population, particularly in those with certain comorbidities, such as Autosomal Dominant Polycystic Kidney Disease (ADPKD). Despite the lack of specific guidelines for stroke prevention in young adults, certain preventive measures can be implemented. Smoking cigarettes is the most significant stroke risk factor in this group. Additionally, psychosocial stress, often exacerbated by academic, career, and financial pressures, is emerging as a modifiable risk factor for stroke in young adults. Key preventive measures include dietary changes, management of underlying health conditions, incorporating regular physical activity into daily routines, smoking cessation, and effective stress management techniques such as mindfulness-based stress reduction (MBSR) and cognitive–behavioral therapy (CBT). Promoting mental health awareness, directing public health campaigns toward young adults, educating them on recognizing stroke symptoms and administering first aid, and improving the quality of healthcare for this population all play a vital role in preventing stroke in young adults.

## 1. Introduction

Stroke is the second most common cause of death worldwide and is the primary cause of long-term physical and cognitive impairments in adults [1,2]. The risk factors for strokes in young adults are generally very similar to traditional risk factors for strokes in the general population. However, there are a couple of risk factors that are pathognomonic for the younger population of adults. Among those are recreational drug use, migraines, oral contraceptive use, pregnancy and post-partum state, and patent foramen ovale [2]. Defining an age cutoff can be challenging and sometimes arbitrary, but previously published studies and registries often classify young adults as those under 50 years of age [3]. The prevalence of stroke varies significantly between the United States and Europe. In the US, young adults account for approximately 10–15% of all strokes, with the incidence of stroke in adults aged 20–44 rising from 17 to 28 per 100,000 people between 1993 and 2015. In Europe, for adults under 55, the incidence of ischemic stroke has increased from 10.7 per 100,000 to 18.1 per 100,000. Globally, the rates of ischemic stroke among young adults differ widely, ranging from as low as 5.8 per 100,000 in central Italy to 97.7 per 100,000 in China [4]. Although there are no specific recommendations or guidelines for primary or secondary stroke prevention in young adults, strokes in this population are largely preventable by addressing modifiable risk factors [3]. Despite older studies suggesting that strokes in younger adults were less severe and had a better prognosis, studies have shown a higher mortality risk in this group, emphasizing the need for further research to improve preventive measures and treatments aimed at reducing stroke risk and improving long-term outcomes for these patients [5].

This emphasizes the urgent need for improved prevention and treatment strategies to mitigate stroke risk and enhance long-term outcomes in this population. 

**Figure 1:**
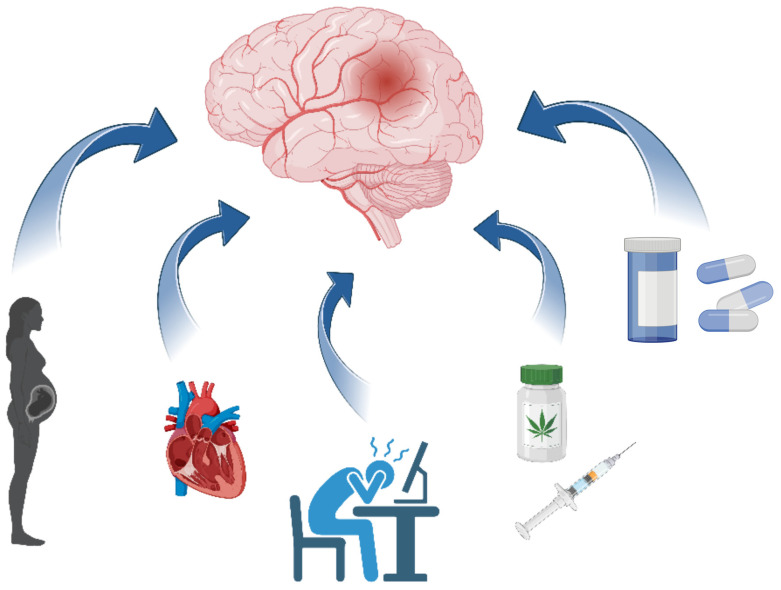
Specific risk factors for stroke in the young population are pregnancy and post-partum state, patent foramen ovale, migraines, recreational drug use, and oral contraceptives.

Figure 1 illustrates specific risk factors that are more prevalent or unique to the young adult population, including pregnancy and the post-partum state, patent foramen ovale, migraines, recreational drug use, and oral contraceptive use. Recognizing and addressing these factors is crucial for effective stroke prevention in this demographic.

## 2. Materials and Methods

This review synthesizes evidence from a wide range of sources, including observational studies, clinical trials, and emerging interventions, to provide a comprehensive overview of stroke prevention in young adults. A narrative review approach was chosen for its flexibility in integrating diverse types of evidence, including epidemiological data, interventional studies, and mechanistic insights. This methodology is particularly well-suited for addressing multifactorial conditions such as stroke, where risk factors, prevention strategies, and therapeutic approaches are continuously evolving.

**Figure 2:**
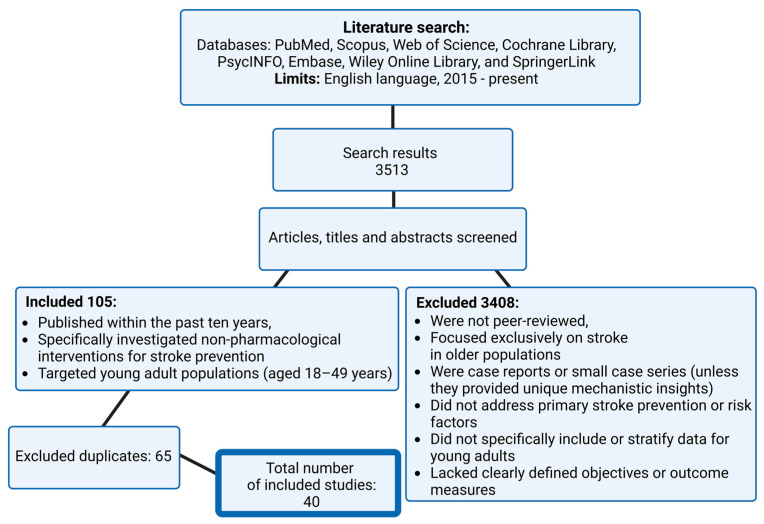
Summarizing the literature search, screening, and selection process for studies included in the review on non-pharmacological stroke prevention in young adults.

A comprehensive search of peer-reviewed English-language literature was conducted across multiple databases, including PubMed, Scopus, Web of Science, Cochrane Library, PsycINFO, Embase, Wiley Online Library, and SpringerLink. The primary search terms encompassed “stroke in young adults”, “stroke risk factors”, and “stroke prevention strategies”. The initial search yielded 3513 studies. After screening titles and abstracts and applying inclusion and exclusion criteria, 105 studies were considered eligible for full-text review. Of these, 40 studies met all inclusion criteria and were ultimately selected for detailed analysis in this review. Inclusion criteria were defined to incorporate studies that (a) were published within the past ten years (2015–2025), with exceptions made for older studies of significant clinical relevance; (b) specifically investigated non-pharmacological interventions for stroke prevention; and (c) targeted young adult populations (aged 18–49 years). Exclusion criteria encompassed studies that (a) were not peer-reviewed; (b) focused exclusively on stroke in older populations; (c) were case reports or small case series, unless they provided unique mechanistic insights; (d) did not address primary stroke prevention or relevant risk factors; (e) did not specifically include or stratify data for young adults; and (f) lacked clearly defined objectives and outcome measures. In cases where multiple similar studies existed, preference was given to systematic reviews or meta-analyses unless more recent primary research offered updated findings. This review synthesizes current knowledge on stroke risk factors in young adults and explores potential non-pharmacological strategies for primary stroke prevention. By integrating findings from existing literature and recent clinical investigations, it critically evaluates established preventive approaches, assesses emerging interventions, and identifies research gaps. Particular emphasis is placed on lifestyle modifications, stress management, and cognitive rehabilitation as key non-pharmacological strategies to mitigate stroke risk in this population. Furthermore, ongoing clinical trials are analyzed to highlight recent advancements and address unmet needs in stroke prevention among young adults. A structured summary of the search results and study selection process is presented in Figure 2. All figures, including the graphical abstract, were created using the BioRender platform (www.biorender.com, accessed on 5 March 2025), to visually represent proposed mechanisms and intervention strategies.

## 3. Overview of Strokes

### 3.1. Definition and Classification

Stroke is an urgent neurological condition caused by an acute focal injury of the central nervous system (CNS) [6,7]. Strokes can be classified into two main categories: ischemic and hemorrhagic, which includes intracerebral hemorrhage and subarachnoid hemorrhage [6,7]. Ischemic strokes represent the majority of acute strokes, occurring by an obstruction of small and large arteries, either by atherosclerosis or cardiac-embolism, or athero-thromboembolism [8]. The other etiology is also a significant cause of ischemic stroke, also in younger patients, like an extracranial and intracranial dissection [8]. These changes result in restricted blood flow to the brain. Intracerebral hemorrhages cause about 15% of acute strokes. They can be deep, lobar, or cerebellar. Most commonly, deep hemorrhages occur from atherosclerosis and untreated arterial hypertension, while lobar hemorrhages usually result from cerebral amyloid angiopathy [8]. Importantly, macrovascular lesions (vascular malformations, cavernomas, aneurysms) can be a significant cause of intracerebral strokes in younger patients [8]. Subarachnoid hemorrhage is, in most cases, caused by a spontaneously ruptured aneurysm, but it can also occur from recreational drug use, coagulopathy, and arteriovenous malformations [9]. Transient ischemic attacks (TIAs) are more common in older adults, but they can occur in younger individuals as well. This is defined as the onset of an episode of focal neurological deficit, resulting from focal cerebral ischemia, with a complete resolution within 24 h since occurrence [10]. A recent study has shown that younger patients who have suffered an ESUS (Embolic Stroke of Undetermined Source) have a significant risk of recurrent stroke/TIA (15% over 15 years) [11]. Lacunar strokes are a subtype of ischemic strokes, representing around 25% of them. They occur due to the obstruction of small penetrating arteries that supply deep brain structures (basal ganglia, thalamus, and internal capsule) [11]. In younger adults, lacunar strokes are often associated with diabetes and hypertension, which are major risk factors for small vascular lesions [12]. In younger adults with diabetes, lacunar strokes are often associated with a higher prevalence of intracranial atherosclerotic stenosis and peripheral vascular disease, reflecting broader manifestations of systemic atherosclerosis [13]. Additional significant etiologies of stroke in this population include both inflammatory and non-inflammatory arteriopathies such as fibromuscular dysplasia, as well as monogenic conditions like Fabry disease and CADASIL [14].

Classic clinical representation of an acute stroke consists of difficulty standing or walking, changes in vision, sudden weakness or droopiness of the face, sudden weakness or numbness of extremities, difficulty speaking, and slurred speech. The most common differential diagnoses that can mimic an acute stroke are seizures, conversion disorder, migraine, and hypoglycemia [15,16]. The classification of strokes is essential for adequate diagnosis and treatment [17]. The TOAST classification system categorizes ischemic strokes based on etiology, which can guide treatment decisions and help predict outcomes [17]. Additionally, numerous specific prognostic scales are commonly used to assess long-term prognosis and outcomes [18]. Proper understanding of classification and clinical presentation of an acute stroke is essential for individualized treatment plan and ensuring the best outcomes for the patient [17,18].

### 3.2. Epidemiology and Risk Factors

Stroke is a very significant global socioeconomic burden, with an incidence of 10.3 million cases per year, as well as 113 million disability adjusted life years (DALY) lost each year [19]. Studies have shown a significant decrease in stroke incidence and mortality in high-income countries, while there have been no notable changes in middle- and low-income countries [20]. Furthermore, a significant growth in disparities has been noted between high-income countries (HIC) and low- and middle-income countries (LMIC), showing a 42% decrease in stroke incidence in HIC and a 100% increase in LMIC [19]. Disparities between different subgroups have been noted too, stroke being more common in men compared to women, and at the same time showing that young and middle-aged women are at higher lifetime risk of stroke compared to men [20]. Given that the majority of acute strokes occur in patients over 50 years of age, it is alarming to note that 10–15% of strokes are reported in the population aged 18–50 [21]. Additionally, the newest research discovered that the global incidence rate of ischemic stroke will rise up to 89.32 per 100,000 population by the year 2030, which further highlights the urgency for effective both prevention and treatment modalities [21]. Timely thrombolytic treatment and mechanical thrombectomy have been shown to significantly improve recovery outcomes in young ischemic stroke patients compared to older individuals [22], further emphasizing the benefits of early intervention in this group.

**Figure 3:**
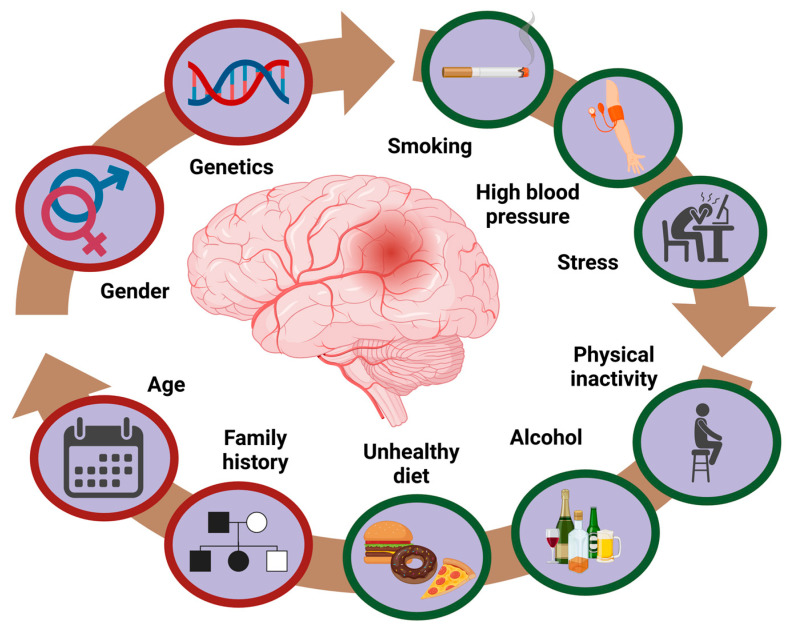
Schematic presentation of modifiable and non-modifiable risk factors for strokes in young adults.

Major stroke risk factors can be categorized as modifiable and non-modifiable [23]. Sex, age, and race are considered non-modifiable, whereas hypertension, smoking, and physical inactivity are among the modifiable risk factors [24]. Other modifiable and non-modifiable risk factors are illustrated in Figure 3. Recent population-based studies in young adults emphasize that modifiable risk factors, particularly low physical activity, hypertension, and smoking, account for the majority of stroke risk in this group. In a large German case-control study, physical inactivity alone explained up to 60% of stroke risk in adults under 55 [25]. Similarly, U.S. data showed that smoking and hypertension were the strongest contributors in White and Black populations, respectively [26]. Hypertension is a very common and significant major risk factor for an acute stroke occurrence, accounting for 25% to 50% of cases [27]. A recent study assessed the risk probability of stroke in patients with hypertension and noted it was very high in both males and females (91% and 70.7%, respectively), and remains high with the persistence of hypertension [28]. Frequent alcohol consumption is a greater risk factor for acute strokes as well. Recent research has shown that moderate and high alcohol intakes are associated with increased odds for stroke, but low intake is not [29]. On the other hand, some older studies indicated that light alcohol consumption can somewhat reduce the risk of a stroke in men, specifically ischemic stroke [30,31]. Risk factors can also be classified as short-term or triggers, such as stress or infections; intermediate-term, such as hypertension and hyperlipidemia; and long-term risk factors—sex, age, and race [24]. Besides the ones mentioned, it is important to note other significant risk factors for stroke: elevated cholesterol levels, diabetes, atrial fibrillation, and obesity, although they are more commonly associated with older populations [32,33].

#### Autosomal Dominant Polycystic Kidney Disease (ADPKD) as a Significant Stroke Risk in Young Adults

Autosomal dominant polycystic kidney disease (ADPKD) is associated with an increased risk of cerebrovascular events, including both ischemic and hemorrhagic strokes, particularly in younger individuals. Studies indicate that patients with ADPKD have a 1.4-fold greater risk of stroke compared to the general population, with this risk being especially pronounced in young adults [34]. Unruptured intracranial aneurysms (UIAs) are significantly more prevalent in this population, with a prevalence ratio of 6.9 compared to individuals without comorbidities [35]. Patients with ADPKD have a markedly increased risk of developing intracranial aneurysms, largely due to impaired vessel wall elasticity and abnormal polycystin expression. These aneurysms frequently occur at arterial bifurcations and are estimated to be 4–5 times more prevalent in ADPKD patients than in the general population [36]. The most relevant contributing factors include early-onset hypertension and the presence of intracranial aneurysms [37,38].

Recent research further emphasizes the neurological burden of ADPKD by highlighting the presence of cerebral microbleeds (CMBs), which are considered markers of small vessel disease and may signal early cerebrovascular injury. A 2025 case-control study reported deep cerebral microbleeds in 20% of individuals with ADPKD, while none were observed in matched controls, even after adjustments for age, sex, and renal function [39]. The results point to a disease-specific susceptibility to microvascular pathology. Such evidence supports the application of advanced neuroimaging modalities, including susceptibility-weighted imaging (SWI) and high-resolution MRI, particularly in symptomatic young adults with ADPKD.

Due to this elevated cerebrovascular risk, screening strategies are essential, especially for early detection of UIAs before rupture. According to current KDIGO 2025 guidelines, magnetic resonance angiography (MRA) or computed tomography angiography (CTA) is recommended for ADPKD patients with a family history of subarachnoid hemorrhage (SAH), those working in high-risk occupations, or individuals undergoing major surgery or pregnancy [38].

Hypertension is the most significant modifiable contributor to stroke in ADPKD and often presents early, sometimes even during adolescence. It becomes more prevalent with age, and studies estimate that up to 60–70% of patients develop hypertension early in the disease course [40,41]. Left ventricular hypertrophy (LVH) is also frequently observed in these patients, even at a young age, and early vascular changes have been reported in normotensive individuals with ADPKD. Furthermore, patients with ADPKD have a higher prevalence of coronary artery aneurysms and cardiac valvular abnormalities compared to the general population [34]. Intensive blood pressure control has been shown to slow cyst growth, preserve renal function, and reduce cardiovascular and cerebrovascular risk. First-line pharmacologic therapy typically includes renin–angiotensin system blockers (e.g., ACE inhibitors or ARBs), with additional lifestyle interventions such as dietary sodium restriction, smoking cessation, and regular aerobic activity recommended to improve outcomes [42]. Interestingly, vascular abnormalities in ADPKD may not be solely driven by hypertension. Polycystins, encoded by the PKD1 and PKD2 genes, are thought to maintain arterial wall integrity. Animal models have shown that loss of polycystins leads to vessel fragility and spontaneous hemorrhage, suggesting a direct role in vascular stability [34]. Additionally, dysregulation of the mTOR pathway—commonly observed in ADPKD due to polycystin-1 dysfunction—may further contribute to vascular remodeling and disease progression [34].

UIAs are a well-documented vascular manifestation of ADPKD, with prevalence rates ranging from 9 to 23%, markedly higher than in the general population. These aneurysms are also more likely to rupture at a younger age, typically around 10 years earlier than in individuals without ADPKD [43]. Subarachnoid hemorrhage (SAH) resulting from aneurysm rupture is one of the most severe complications in ADPKD, representing a major cause of neurological morbidity in these patients [36]. Individuals with a family history of aneurysmal SAH have a particularly high risk of developing intracranial aneurysms. Siblings of affected patients have been shown to have an increased risk for both unruptured aneurysms and SAH, further supporting the need for targeted screening [44]. Additionally, postmenopausal women may be at increased risk due to the decline of estrogen’s vascular protective effects [36].

In addition to guideline-based recommendations, some experts advocate broader screening strategies. A recent study identified a 5% prevalence of UIAs in individuals aged 35 and older with hypertension and a history of smoking. Although only a small proportion of these aneurysms required preventive intervention, the findings highlight the potential benefits of targeted screening in selected subgroups [45]. Screening can help identify small aneurysms that may not require immediate treatment but should be followed longitudinally through imaging surveillance due to their potential for future growth or rupture [36]. Despite these clear associations, many young adults with ADPKD remain unscreened for cerebrovascular risk, particularly in primary care settings [46]. While recent studies have supported universal screening as a strategy that may increase quality-adjusted life years, others caution that such an approach may not be universally appropriate due to risks of overdiagnosis, psychological burden, healthcare costs, and variability in available expertise [36].

Given the rising incidence of stroke among young adults—a group often underrepresented in stroke-related research—early identification and targeted prevention of high-risk conditions like ADPKD are essential.

### 3.3. Screening and Early Detection

Many young adults with stroke may not recognize early warning signs or seek medical attention promptly, contributing to delays in diagnosis and treatment. This population often underestimates their risk for vascular disease, leading to missed opportunities for prevention and early intervention [47]. This low-risk perception among younger adults presents a significant barrier to timely diagnosis and prevention efforts, often resulting in delayed care and missed opportunities for intervention [48]. Numerous screening tests are used for stroke detection, but specific data on their effectiveness in young adults are still limited. Studies have shown that the most commonly misdiagnosed acute stroke in this population is posterior circulation stroke [49]. One reason for this misdiagnosis may be the use of the FAST (Face, Arm, Speech, Time) screening test, which is less sensitive to posterior strokes and often leads to delayed diagnosis and treatment. A recent study demonstrated that FAST missed 38% of posterior circulation strokes [50]. It is recommended to use additional diagnostic tools, such as MRI, to improve early detection of posterior circulation strokes in young adults, which could significantly improve outcomes and help reduce long-term disabilities in these patients [51].

## 4. Clinical Studies on Stroke in Young Adults: Treatment and Outcomes

Multiple studies and randomized controlled trials have contributed to a more nuanced understanding of stroke in young adults, emphasizing both the effectiveness of standard acute-phase therapies and the importance of age-specific prevention and rehabilitation approaches (Table 1).

A large national registry demonstrated that endovascular thrombectomy is a safe and effective option for younger patients with large vessel occlusion, with 61% achieving functional independence at 90 days and significantly lower mortality (7%) compared to older adults (32%) [52]. The more favorable outcomes in this group may be partly explained by lower comorbidity rates and increased neuroplastic potential.

Intravenous thrombolysis with alteplase (rtPA) has also shown strong efficacy in younger individuals. In a retrospective analysis of patients aged 16 to 49, none died during a three-month follow-up period, and a majority reached favorable functional outcomes [53]. This supports the early use of thrombolytic therapy in this demographic and confirms the relevance of standard acute-phase treatments, provided they are administered without delay.

When it comes to lifestyle factors, the connection between marijuana use and ischemic stroke has been explored in a population-based case-control study including over 2000 individuals aged 15 to 49. After controlling for common confounders such as tobacco use and hypertension, no significant association was found. However, a trend toward higher risk was observed among those using marijuana weekly or more often [54]. While not conclusive, this points to a possible dose–response relationship that warrants further investigation.

Preventive strategies tailored to younger populations have also been the focus of recent interventions. One theory-driven, nurse-led program developed for young African American adults incorporated multimedia education, personalized risk assessment, and behavioral self-monitoring over six weeks. Participants reported enhanced risk perception and adopted healthier habits, including improved diet and increased physical activity [55]. Such culturally responsive approaches show promise in addressing modifiable risk factors in vulnerable subgroups.

Rehabilitation research has begun to move beyond physical recovery, recognizing the unique psychosocial impact of stroke in younger adults. A structured, skills-based program focusing on narrative work and coping strategies led to improvements in self-efficacy, emotional well-being, and community reintegration [56]. In addition, expressive arts-based therapy, involving activities such as visual arts and group reflection, produced measurable improvements in emotional resilience, social support, and spiritual well-being, alongside reductions in stress markers like salivary cortisol [57]. These integrative models reflect a broader, more holistic view of recovery [57].

Despite these advances, stroke in young adults continues to be under-recognized in acute care settings. A recent consensus statement noted that up to 15% of ischemic strokes occur in those under 50, yet atypical presentations such as isolated dizziness or headache often led to diagnostic delays [49]. This highlights the need for increased awareness among clinicians and the development of age-specific diagnostic protocols that better capture the variability of stroke presentations in this population [49].

Taken together, these findings point to several key conclusions. First, young adults clearly benefit from standard reperfusion therapies when diagnosed in a timely manner, underscoring the need for clinical vigilance in emergency settings. Second, lifestyle interventions and targeted education appear particularly effective in reducing stroke risk in this group, especially when adapted to cultural and social contexts. Third, the long-term psychosocial consequences of stroke in young adults call for integrative rehabilitation strategies that extend beyond physical therapy to include mental health support, identity recovery, and community reintegration.

Altogether, current evidence reinforces that stroke in young adults is not simply a milder form of the disease seen in older individuals. Unique risk factors, recovery patterns, and psychosocial outcomes define this group as a distinct clinical category. To improve both immediate and long-term outcomes, future efforts must focus on tailored diagnostics, culturally sensitive prevention, and comprehensive rehabilitation strategies that reflect the realities of younger stroke survivors.

### 4.1. Comparative Insights: Stroke in Younger vs. Older Adults

Age-based comparisons of stroke incidence, clinical presentation, risk profiles, and outcomes have revealed substantial differences between younger and older adults, underscoring the need for more individualized approaches across the care continuum.

Over the past two decades, stroke incidence has shifted considerably. While rates have declined among adults over 55, a marked increase has been observed in younger populations. Between 2002 and 2018, ischemic stroke rose by 67% in individuals under 55, contrasting with a 15% decrease in older adults [58]. The upward trend in this age group appears closely tied to the rising prevalence of modifiable risk factors such as hypertension, obesity, and type 2 diabetes conditions that were once more commonly associated with older patients [58].

Symptom variability adds complexity to clinical assessment in early-onset stroke. In one hospital-based analysis, adults aged 18 to 50 more frequently presented with fluctuating or progressive neurological deficits compared to those over 50 [59]. Atypical symptoms, including isolated headache, dizziness, or seizure, often lead to misdiagnosis or delays in care. Given the narrower diagnostic lens often applied in younger individuals, emergency settings must adapt to recognize early signs more effectively [59].

Differences in comorbidities and inpatient outcomes have also been documented. Older adults with stroke typically present with higher rates of atrial fibrillation, prior cerebrovascular events, and chronic hypertension, all of which contribute to elevated in-hospital mortality and greater dependence at discharge [60]. In contrast, younger patients, though generally healthier, still face considerable functional challenges. Their likelihood of achieving independence at discharge, however, tends to be higher, likely due to better baseline health, shorter time to treatment, and greater neuroplasticity [60].

Stroke subtype and underlying etiology also vary with age. In individuals under 50, lacunar and cryptogenic strokes are more frequently encountered, while strokes due to large artery atherosclerosis or cardio-embolism are more common in older patients [61]. These differences point to the importance of age-adapted diagnostic approaches and tailored secondary prevention strategies.

Large-scale hospitalization data from the United States further support the growing significance of stroke in younger adults. Among more than four million ischemic stroke admissions recorded between 2003 and 2014, nearly 5% involved individuals aged 18 to 45 [62]. Alongside traditional cardiovascular risk factors, a wide range of non-conventional contributors, including HIV infection, autoimmune disease, and congenital heart defects, was associated with stroke in this group [62]. Such complexity highlights the need for comprehensive screening protocols that go beyond standard cardiovascular parameters.

Findings from sub-Saharan Africa offer an important global perspective. In a Tanzanian cohort, one-third of stroke patients were aged 45 or younger, with a higher prevalence of hemorrhagic stroke among younger individuals compared to older counterparts [63]. Key risk factors included undiagnosed hypertension, HIV infection, hormonal contraceptive use, elevated LDL cholesterol, and sickle cell disease. Despite high overall 30-day mortality, individuals in early adulthood had comparatively better survival outcomes [63]. These observations reflect the dual burden of communicable and non-communicable conditions and point to the importance of integrating stroke risk assessment into broader public health strategies, particularly in resource-limited settings. Similar patterns emerged in another retrospective analysis, where nearly one in five stroke patients were aged 18 to 45 [64]. Hemorrhagic events were again more prevalent among younger adults (21.5% vs. 11.6%, *p* = 0.011). While hypertension was the leading risk factor across all ages, diabetes mellitus and dyslipidemia were also present in the younger group, though to a lesser extent than in older individuals [64]. Their findings suggest that vascular risk accumulates earlier than often assumed, making a strong case for preventive strategies such as early cardiovascular screening and lifestyle intervention beginning in early adulthood. A comparative overview of these studies is presented in Table 2, highlighting key differences in stroke incidence, clinical features, risk factor profiles, and outcomes between younger and older adults.

Taken together, these studies demonstrate that stroke in younger adults is not a milder version of the disease seen in older populations but a distinct clinical entity. From differences in pathophysiology and risk exposure to variation in clinical presentation and recovery trajectories, early-onset stroke demands a differentiated clinical and public health response. Tailored diagnostic frameworks, targeted prevention efforts, and rehabilitation strategies that account for social, vocational, and psychological realities are critical to improving long-term outcomes in this growing patient population.

### 4.2. Comparative Summary of Non-Pharmacological Strategies in Young Adults

In addition to clinical and psychosocial interventions, various non-pharmacological strategies have been proposed to reduce stroke risk in young adults. Table 3 summarizes the most relevant findings from studies investigating the effects of dietary patterns, physical activity, stress reduction, educational programs, and behavioral counseling in this population. The comparative format allows visualization of outcomes across diverse populations and study types, highlighting which strategies may be most effective for specific subgroups.

## 5. Dietary and Lifestyle Modifications

Recent research highlights the significant role of nutrition in stroke prevention. Diets rich in fruits, vegetables, whole grains, legumes, nuts, and unsaturated fats—such as olive oil—have been consistently associated with a significantly reduced risk of stroke [65,73]. Supporting this, a recent international review confirmed that poor dietary practices, including high consumption of saturated fats and refined sugars, are frequently observed among young stroke patients. These findings underscore the importance of implementing age-specific nutritional counseling as part of primary prevention strategies in younger adults [74]. Furthermore, adherence to a Mediterranean-style diet has been linked to reduced stroke incidence [75]. Fiber-rich foods, particularly legumes and whole grains, help regulate blood pressure and postprandial glucose levels, thereby contributing to stroke risk reduction. In contrast, the consumption of saturated fats—commonly found in processed foods, red meat, and full-fat dairy products—should be minimized, as these can raise cholesterol levels and promote atherosclerosis [76]. In addition, hyperlipidemia, a diet-related metabolic condition, has been found to be significantly associated with ischemic stroke in young adults, reinforcing the importance of early dietary interventions and lipid management [77]. Despite earlier assumptions, studies have shown no clear association between higher intake of vitamins E and C and reduced stroke incidence [78,79].

The relationship between alcohol consumption and stroke risk remains complex. While moderate intake—particularly of red wine—may confer certain cardiovascular benefits, excessive alcohol use is clearly associated with an increased risk of all stroke subtypes, especially intracerebral hemorrhage. The net effect of alcohol may depend on regional consumption patterns, accompanying lifestyle behaviors, and coexisting risk factors [29]. In young adults, episodic heavy drinking has been identified as a notable risk contributor, with a population-attributable risk of 10.2%, reinforcing the need for alcohol-focused interventions in this demographic [25]. Some potential protective effects of red wine have been attributed to specific polyphenolic compounds with disease-modifying properties [80].

Excessive dietary salt intake is another modifiable factor contributing to both ischemic and hemorrhagic strokes [81]. Even modest reductions in salt intake have been linked to meaningful improvements in cardiovascular health. Among individuals with hypertension, average reductions of 7 mm Hg in systolic and 4 mm Hg in diastolic blood pressure have been observed. In those with resistant hypertension, the effects are even more pronounced—up to 23 mm Hg systolic and 9 mm Hg diastolic [82].

Psychosocial stress is also associated with elevated stroke risk. However, studies indicate that individuals with a greater perceived locus of control—both at work and at home—experience a significantly lower stroke incidence. This suggests that internal resilience and perceived agency may buffer the physiological effects of chronic stress [66]. In parallel, recent data indicate that younger adults are more likely to adopt dietary patterns that at least partially align with established stroke prevention recommendations. Although these findings are not exclusive to the young, they underscore the broader importance of diet quality in reducing stroke risk across all age groups [67].

Beyond individual habits, structured lifestyle intervention programs have shown promise as part of comprehensive prevention strategies for stroke in younger adults. One such example is a randomized pilot study conducted in primary care settings among adults aged 45 to 75 years with moderate-to-high stroke risk. The intervention promoted “engaging everyday activities” (EEAs) to encourage physical activity and reduce sedentary behavior. Although not powered to detect changes in stroke incidence, the program improved participants’ lifestyle behaviors and demonstrated feasibility in real-world clinical practice [68].

These findings highlight the growing importance of not only educating younger adults about healthy lifestyle choices but also developing accessible, real-world programs that support sustainable behavior change.

## 6. Physical Activity Increase

Regular physical activity provides well-established cardiovascular benefits and plays a critical role in mitigating key stroke risk factors such as hyperlipidemia, obesity, and atherosclerosis [83]. Recent prevention initiatives specifically targeting younger adults have identified insufficient physical activity as a major modifiable behavioral risk factor, reinforcing the need for movement-focused interventions in this population [74].

Data from a case-control study revealed that physical inactivity represented the single most significant contributor to stroke risk in young adults, with a population-attributable risk (PAR) of 59.7% [25]. Elevated risk was evident even among individuals under the age of 35, pointing to the importance of early lifestyle habits in stroke pathogenesis [25]. Findings from a large multicenter study further confirmed the widespread nature of inactivity among young stroke patients. Nearly half (48.2%) of those with ischemic stroke or transient ischemic attack (TIA) reported low levels of physical activity [69]. The prevalence was higher in women (52.7%) than in men (44.1%) and reached its peak in individuals under 35, where 56.5% were inactive [69]. These patterns indicate a particular vulnerability among younger women and suggest that future interventions should incorporate both age- and sex-specific strategies to effectively address physical inactivity in stroke prevention.

A recent meta-analysis has shown that both moderate and high levels of physical activity are associated with a reduced risk of stroke [84]. Subsequent studies have reinforced these findings, recommending at least 40 min of moderate-to-vigorous aerobic exercise, three to four times per week, as an effective preventive measure. While men appear to benefit more from moderate-intensity activity, women tend to derive greater benefit from lower-intensity forms of exercise [85].

Higher levels of leisure-time physical activity (LTPA) are consistently associated with a lower incidence of stroke. Even lower levels of LTPA—often considered insufficient by standard guidelines—have been shown to contribute to stroke risk reduction [86]. Moreover, increased LTPA is linked to better functional outcomes, including a reduced risk of death or dependency in activities of daily living (ADL) within three months post-stroke [87]. Regular engagement in sports and structured exercise has also been strongly associated with reduced stroke risk, further emphasizing the importance of maintaining an active lifestyle, particularly in younger populations [88,89].

Complementing these findings, a longitudinal cohort study from Sweden demonstrated that excessive body mass index (BMI) gain during adolescence—spanning puberty through early adulthood—is significantly associated with an elevated incidence of stroke by midlife. This effect appears to be mediated, at least in part, by the early development of hypertension [90].

Taken together, these findings strongly support the inclusion of daily physical activity and healthy weight maintenance as central components of stroke prevention strategies targeting young adults.

## 7. Stress Reduction

Psychosocial stress is a modifiable risk factor for stroke [66]. Although the exact mechanisms remain unclear, research has demonstrated that psychosocial stress significantly increases stroke risk in adults [91]. It contributes to inflammation in blood vessels, oxidative stress, and immune system dysregulation, all of which play crucial roles in the pathophysiology of stroke [91]. Early career stress, marked by financial concerns and job-related pressures, has also been linked to heightened psychological distress, further increasing the risk of various health complications [92].

Interventions aimed at reducing stress, such as mindfulness-based stress reduction (MBSR) and cognitive–behavioral therapy (CBT), have shown promising results in alleviating stress and improving cardiovascular health. Mindfulness practices, in particular, have been found to reduce inflammatory markers and improve psychological well-being, potentially lowering stroke risk by addressing both the mental and physical components of stress [70]. MBSR and CBT help regulate autonomic nervous system activity, reduce sympathetic nervous system hyperactivity, and enhance parasympathetic function, all of which can improve cardiovascular health. Furthermore, these interventions have been shown to lower cortisol levels, a key stress hormone, and improve endothelial function, which may directly influence stroke risk. By enhancing emotional regulation and reducing negative health behaviors associated with stress, MBSR and CBT offer effective approaches to lowering stroke risk [71,93].

## 8. Cessation of Smoking

Tobacco smoking remains one of the most significant and preventable risk factors for stroke in young adults. A large case-control analysis estimated that smoking contributes to approximately 20–30% of all strokes in this age group, with prevalence rates notably higher among younger stroke patients compared to older individuals [26]. Furthermore, data from a European study of adults aged 18–55 indicated that smoking alone accounted for nearly 25% of stroke cases, with a population-attributable risk (PAR) of 27.1% among men [25].

Emerging evidence highlights recreational drug use, particularly cannabis, as another relevant modifiable risk factor in this population. A 2024 systematic review and meta-analysis of six studies involving over 119 million participants found that cannabis abuse was associated with a modest but statistically significant increase in stroke risk among young adults (adjusted odds ratio ≈ 1.21 after adjusting for smoking and alcohol use) [94].

In addition to its established association with stroke, smoking is a well-recognized high-risk behavior that contributes to cardiovascular disease, multiple forms of cancer, and a range of neurological disorders [95]. Among young adults with stroke, tobacco use is consistently identified as a prominent and preventable risk factor [96]. As a modifiable behavior, smoking plays a central role in both primary and secondary stroke prevention. Evidence indicates that cessation substantially lowers the risk of recurrent vascular events and all-cause mortality [97]. Data from recent research reveal a dose-dependent relationship between cigarette consumption and ischemic stroke risk in young men, suggesting that even smoking reduction—rather than complete cessation—may yield measurable benefits [91]. Despite this, only about half of stroke survivors report quitting or cutting back following the event. Those with coexisting conditions such as depression, anxiety, or alcohol misuse are significantly less likely to change their smoking behavior [86]. Although the benefits of cessation are well documented, overall quit rates remain suboptimal. This may be due, in part, to the limited use of available resources, including evidence-based counseling strategies, behavioral interventions, and pharmacologic therapies aimed at supporting cessation [72,98].

## 9. Mental Health Improvement

A stable and good mental state has been shown to be a positive factor in reducing stroke risk, with higher levels of emotional vitality strongly correlating with a lower risk of stroke [99]. Mental health disorders, such as depression, are more prevalent in younger stroke patients compared to older patients and may contribute to delayed care-seeking behavior or reduced engagement in preventive measures [25,40]. Furthermore, studies clearly indicate that young stroke survivors are at a significantly higher risk of developing new-onset anxiety and depression compared to older individuals. Specifically, those under the age of 50 are nearly three times more likely to experience anxiety and depression than their older counterparts [100]. Additionally, preventive therapies for depression following stroke have proven to be both effective and safe in reducing post-stroke depression [101]. Given the increased vulnerability of young stroke survivors, physician advice plays a crucial role in secondary prevention for this age group. It has been shown to significantly improve physical activity, which in turn can reduce the risk of further vascular events in younger populations [102]. Research indicates that recreational drug use significantly increases the risk of stroke among young adults. A study from Kasr Al-Ainy Hospital reported that 41% of young stroke patients had substance use disorders. Heroin use disorder was linked to higher mortality, while alcohol, heroin, and cocaine use disorders were associated with poorer stroke outcomes [103]. These findings highlight the importance of counseling young patients on the dangers of recreational drug use as a part of stroke prevention strategies.

## 10. Public Health Initiatives and Education

The primary objective of stroke prevention is to reduce incidence, long-term disability, and mortality, particularly among younger adults. Achieving this goal requires effective identification, reduction, and management of modifiable risk factors. Although stroke care has not always been a public health priority, the foundational functions of public health—assessment, policy development, and assurance—can enable health agencies to play a central role in improving care quality for stroke and cardiovascular patients [104]. According to the Centers for Disease Control and Prevention (CDC), up to 80% of strokes are preventable through strategies such as diabetes prevention, smoking cessation, cholesterol and blood pressure control, use of anticoagulants in individuals with atrial fibrillation, and reduced alcohol consumption [105].

School-based educational programs have demonstrated the potential for early prevention. One recent initiative targeting high school students focused on increasing knowledge about stroke prevention, symptom recognition, and emergency response. Knowledge surveys conducted before and after the program revealed significant improvement, with the highest scores observed among students who also took on teaching roles. This peer-teaching approach served as a strong motivator for engagement and retention of information [106].

Despite the promise of such programs, broader implementation often faces real-world challenges. For instance, a survey conducted among African American communities found that participants with higher educational attainment displayed greater awareness of stroke risk factors. However, barriers such as psychosocial stress and financial strain were frequently cited as impediments to prevention. Many respondents also preferred receiving health information in clinical rather than community-based settings [107]. Digital tools have also been explored as accessible and scalable public health solutions. In a multi-country survey across four Arab nations, a brief three-minute online video led to statistically significant improvements in stroke-related knowledge, including recognition of risk factors and warning signs, as well as awareness of the need for prompt medical response. Participants, most of whom were under 55 and held university degrees, cited the visual design, simplicity of language, and cultural relevance as key factors in the video’s effectiveness [108]. Mass media campaigns can also play a role in increasing public awareness. In Ireland, the National Stroke Awareness Campaign (Act F.A.S.T.) was associated with improved recognition of stroke symptoms. Among those surveyed 18 months after the campaign, 93% recalled having seen the message, and over 80% were able to correctly identify stroke warning signs. Although only a third remembered the name or slogan of the campaign, more than half stated they would go directly to the hospital upon noticing symptoms—an important behavioral shift [109].

While educational efforts clearly improve awareness, they are often insufficient to produce sustained behavior change or reduce recurrence rates when used alone. Current AHA/ASA guidelines recommend patient education as a component of care but acknowledge that it does not consistently lead to measurable improvements in health behaviors. Greater success has been observed in randomized controlled trials that combine education with physical activity, dietary modification, and individualized counseling. In one trial involving 60 participants, a combination of lifestyle interventions resulted in significant reductions in recurrent stroke or TIA (RR 0.23, 95% CI: 0.07–0.72) and lower all-cause mortality (RR 0.11, 95% CI: 0.01–1.98) over 3.5 years of follow-up. Another trial from Japan, which included 70 participants and integrated exercise, salt restriction, and nutritional counseling, was stopped early due to a significant reduction in the combined outcome of stroke-related mortality and cardiovascular hospitalizations after a median follow-up of 2.9 years [110].

Evidence suggests that lifestyle interventions are most effective when they are interactive, tailored to the individual, and delivered by trained healthcare professionals with sufficient time and resources. However, systematic reviews indicate that the optimal model for reducing recurrent stroke through lifestyle-based strategies is still not well established [110].

For educational initiatives to have a sustained impact, public health efforts should be adaptive, community-informed, and grounded in a multifaceted approach. Integrating clinical counseling, community engagement, and digital technologies offers the best opportunity to extend reach and improve the long-term effectiveness of stroke prevention programs.

## 11. Conclusions

Although stroke is less common in young adults, its rising incidence and lasting consequences demand greater clinical and research attention. Unique risk factors in this group, such as recreational drug use, oral contraceptives, and chronic stress, are often under-recognized in current prevention models. Inadequate age-specific screening and prevention guidelines contribute to diagnostic delays and missed opportunities for intervention.

To improve outcomes, future research should focus on developing tailored prevention strategies and evaluating the efficacy of non-pharmacological interventions, such as mindfulness and structured lifestyle programs, through rigorous clinical trials. Despite efforts, many existing RCTs on secondary prevention in high-risk groups show limited success, underscoring the need for more effective, context-sensitive approaches. Addressing upstream social determinants like housing instability, food insecurity, and limited access to care is essential for reducing cardiovascular risk and improving treatment adherence. Integrating social needs assessments into clinical practice may help identify at-risk individuals and guide comprehensive support.

Finally, public health initiatives must include culturally sensitive, age-appropriate education on stroke and mental health, helping young adults recognize risk factors and seek timely care. Aligning prevention efforts with the lived realities of this population is crucial for reducing stroke burden and improving long-term outcomes.

## Figures and Tables

**Table 1:** Key clinical studies on strokes in young adults: treatments and outcomes.

Study (Author, Year)	Study Design	Sample Size	Key Findings	Limitations
Brouwer et al. (2022) [52]	Prospective registry (MR CLEAN)	3256 (310 aged 18–49)	Endovascular thrombectomy: 61% of young patients achieved mRS 0–2 at 90 days; mortality was lower than older group (7% vs. 32%)	Observational design; no control group
Putaala et al. (2009) [53]	Retrospective cohort	48 young adults	IV thrombolysis yielded excellent 3-month outcomes; no deaths reported	Small sample size; retrospective bias
Dutta et al. (2021) [54]	Population-based case-control	2242 total (1090 cases, 1152 controls)	No significant association between marijuana use and early ischemic stroke	Self-reported exposure; residual confounding
Aycock et al. (2023) [55]	Feasibility RCT	30 African American young adults	Improved stroke risk perception and health behaviors via SCORRE intervention	Pilot nature; short duration and small cohort
Lo et al. (2021) [56]	RCT protocol	Target: 208	Narrative-based skills-building aims to improve reintegration and self-efficacy	Results pending; study ongoing
Chan et al. (2021) [57]	RCT	Target: 154	Expressive arts therapy improved mood, stress, and QoL in young survivors	Generalizability limited; long-term outcomes not yet assessed
Singhal et al. (2013) [49]	Expert consensus	N/A	10–15% of ischemic strokes occur in young adults; highlighted diagnostic delays	No original data; relies on expert opinion

**Table 2:** Comparison of stroke characteristics, risk factors, and outcomes between younger and older adults across key clinical studies.

Study (Author, Year)	Population	Comparison Focus	Younger Adults	Older Adults
Li et al., 2022 [58]	National registry data, USA (2002–2018)	Incidence trend	↑ 67% ischemic stroke incidence under 55	↓ 15% incidence in ≥55
Huggins et al., 2020 [59]	432 stroke inpatients (18–50 vs. >50)	Symptom presentation	43% had fluctuating/ progressive symptoms	27% had fluctuating/ progressive symptoms
Fonarow et al., 2010 [60]	>500,000 ischemic stroke admissions	Comorbidities and short-term outcomes	Fewer comorbidities; lower in-hospital mortality; better functional recovery	More AF, prior stroke, HTN; higher mortality
Lutski et al., 2017 [61]	Ischemic stroke registry	Functional outcome and etiology	70% mRS ≤ 2; more lacunar/cryptogenic strokes	Lower favorable outcome; more atherosclerosis/cardio-embolism
Patel et al., 2021 [62]	4.2M AIS admissions (USA, 2003–2014)	Risk factors	More obesity, drug use, HIV, congenital heart disease, autoimmune disorders	More traditional risk factors (HTN, AF, CA)
Matuja et al., 2020 [63]	369 stroke patients in Tanzania	Risk profile and mortality	42.3% hemorrhagic stroke; HTN, HIV, contraception, sickle cell, LDL ↑; 49.1% 30-day mortality	27.2% hemorrhagic stroke; 67.2% 30-day mortality
Moosa et al., 2021 [64]	513 stroke patients in Bahrain	Stroke type and comorbidities	21.5% hemorrhagic stroke; HTN (43%), DM (24.7%), DL (16.1%)	11.6% hemorrhagic; HTN (74%), DM (63%), DL (32.2%)

**Table 3:** Comparative summary of non-pharmacological interventions and outcomes related to stroke prevention or recovery in young adults.

Study (Author, Year)	Intervention	Population	Key Findings	Notes
Aycock et al., 2023 [55]	Nurse-led Health Education	Young African American adults (18–45)	Improved stroke risk perception, increased health behavior adherence	RCT; population-specific
Chan et al., 2021 [57]	Expressive Arts Therapy	Young stroke survivors (Chinese cohort)	Improved mental health, emotional expression and quality of life	RCT; post-stroke focus
Spence, 2018 [65]	Mediterranean-style Diet	General population; data cited for young adults	Associated with significantly reduced stroke incidence in observational studies	Not age-stratified; observational data
Reddin et al., 2022 [66]	Stress Reduction/Perceived Control	INTERSTROKE population (includes young adults)	Higher locus of control associated with lower stroke risk	Large-scale observational study
Sobalska et al., 2020 [67]	Dietary Pattern Assessment	Polish young adults (20–45)	Moderate adherence to preventive diets	Cross-sectional; context-dependent
Mälstam et al., 2023 [68]	“Make My Day” Lifestyle Program	Adults aged 45–75 (includes younger subgroup)	Improved physical activity and reduced sedentary behavior	Pilot RCT; not powered to assess stroke incidence
von Sarnowski et al., 2013 [69]	Physical Activity (Leisure-Time)	Young adults (<55), n = 5023	Physical inactivity PAR = 59.7%; most prevalent in women <35	Case-control; specific to young adults
Loucks et al., 2015 [70]	Mindfulness-Based Stress Reduction (MBSR)	Young and middle-aged adults with CV risk	Reduced inflammatory markers; improved autonomic function	Mixed-methods; theoretical and empirical basis
Calderone et al., 2024 [71]	Cognitive–Behavioral Therapy (CBT)	Young adults (pilot study)	Reduced cortisol levels, improved cardiovascular health indicators	Pilot RCT; small sample size
Markidan et al., 2018 [72]	Smoking Cessation Counseling	Young men	Dose-dependent reduction in ischemic stroke risk after quitting	Large population-based study

## References

[1] Johnson C.O., Nguyen M., Roth G.A., Nichols E., Alam T., Abate D., Abd-Allah F., Abdelalim A., Abraha H.N., Abu-Rmeileh N.M. (2019). Global, Regional, and National Burden of Stroke, 1990–2016: A Systematic Analysis for the Global Burden of Disease Study 2016. Lancet Neurol..

[2] Bukhari S., Yaghi S., Bashir Z. (2023). Stroke in Young Adults. J. Clin. Med..

[3] Smajlović D. (2015). Strokes in Young Adults: Epidemiology and Prevention. Vasc. Health Risk Manag..

[4] Yahya T., Jilani M.H., Khan S.U., Mszar R., Hassan S.Z., Blaha M.J., Blankstein R., Virani S.S., Johansen M.C., Vahidy F. (2020). Stroke in Young Adults: Current Trends, Opportunities for Prevention, and Pathways Forward. Am. J. Prev. Cardiol..

[5] Rutten-Jacobs L.C., Arntz R.M., Maaijwee N.A., Schoonderwaldt H.C., Dorresteijn L.D., van Dijk E.J., de Leeuw F.E. (2013). Long-Term Mortality after Stroke among Adults Aged 18 to 50 Years. JAMA.

[6] Dopler B. (2023). Stroke Prevention. Del. J. Public Health.

[7] Sacco R.L., Kasner S.E., Broderick J.P., Caplan L.R., Connors J.J.B., Culebras A., Elkind M.S.V., George M.G., Hamdan A.D., Higashida R.T. (2013). An Updated Definition of Stroke for the 21st Century. Stroke.

[8] Murphy S.J., Werring D.J. (2020). Stroke: Causes and Clinical Features. Medicine.

[9] Tadi P., Lui F. (2024). Acute Stroke. StatPearls.

[10] Lioutas V., Ivan C.S., Himali J.J., Aparicio H.J., Leveille T., Romero J.R., Beiser A.S., Seshadri S. (2021). Incidence of Transient Ischemic Attack and Association with Long-Term Risk of Stroke. JAMA.

[11] Nassif M., Annink M.E., Yang H., Rettig T., Roos Y., van den Brink R., Tijssen J., Mulder B., de Winter R.J., Bouma B.J. (2021). Long-Term (>10-Year) Clinical Follow-up after Young Embolic Stroke/TIA of Undetermined Source. Int. J. Stroke.

[12] Gore M., Bansal K., Khan S.M.Z., Lui F., Asuncion R.M.D. (2024). Lacunar Stroke. StatPearls.

[13] Palacio S., McClure L.A., Benavente O.R., Bazan C., Pergola P., Hart R.G. (2014). Lacunar Strokes in Patients with Diabetes Mellitus: Risk Factors, Infarct Location, and Prognosis. Stroke.

[14] Katsnelson M.J., Della-Morte D., Rundek T. (2012). Stroke in young. Period. Biol..

[15] Yew K.S., Cheng E.M. (2015). Diagnosis of Acute Stroke. Am. Fam. Physician.

[16] Andersson J., Rejnö Å., Jakobsson S., Hansson P.-O., Nielsen S.J. (2024). Symptoms at Stroke Onset as Described by Patients: A Qualitative Study. BMC Neurol..

[17] Adams H.P., Bendixen B.H., Kappelle L.J., Biller J., Love B.B., Gordon D.L., Marsh E.E. (1993). Classification of Subtype of Acute Ischemic Stroke: Definitions for Use in a Multicenter Clinical Trial. TOAST. Trial of Org 10172 in Acute Stroke Treatment. Stroke.

[18] Drozdowska B.A., Singh S., Quinn T.J. (2019). Thinking About the Future: A Review of Prognostic Scales Used in Acute Stroke. Front. Neurol..

[19] Pandian J.D., Gall S.L., Kate M.P., Silva G.S., Akinyemi R.O., Ovbiagele B.I., Lavados P.M., Gandhi D.B.C., Thrift A.G. (2018). Prevention of Stroke: A Global Perspective. Lancet.

[20] Guzik A., Bushnell C. (2017). Stroke Epidemiology and Risk Factor Management. Continuum.

[21] Saini V., Guada L., Yavagal D.R. (2021). Global Epidemiology of Stroke and Access to Acute Ischemic Stroke Interventions. Neurology.

[22] Stefanovic Budimkic M., Pekmezovic T., Beslac-Bumbasirevic L., Ercegovac M., Berisavac I., Stanarcevic P., Padjen V., Jovanović D.R. (2017). Long-Term Prognosis in Ischemic Stroke Patients Treated with Intravenous Thrombolytic Therapy. J. Stroke Cerebrovasc. Dis..

[23] Pu L., Wang L., Zhang R., Zhao T., Jiang Y., Han L. (2023). Projected Global Trends in Ischemic Stroke Incidence, Deaths and Disability-Adjusted Life Years from 2020 to 2030. Stroke.

[24] Boehme A.K., Esenwa C., Elkind M.S. (2017). Stroke Risk Factors, Genetics, and Prevention. Circ. Res..

[25] Aigner A., Grittner U., Rolfs A., Norrving B., Siegerink B., Busch M.A. (2017). Contribution of Established Stroke Risk Factors to the Burden of Stroke in Young Adults. Stroke.

[26] Aradine E.M., Ryan K.A., Cronin C.A., Wozniak M.A., Cole J.W., Chaturvedi S., Dutta T.L.M., Hou Y., Mehndiratta P., Motta M. (2022). Black-White Differences in Ischemic Stroke Risk Factor Burden in Young Adults. Stroke.

[27] Gorelick P.B., Whelton P.K., Sorond F., Carey R.M. (2020). Blood Pressure Management in Stroke. Hypertension.

[28] Li A., Ji Y., Zhu S., Hu Z.-H., Xu X.-J., Wang Y.-W., Jian X.-Z. (2022). Risk Probability and Influencing Factors of Stroke in Followed-up Hypertension Patients. BMC Cardiovasc. Disord..

[29] Smyth A., O’Donnell M., Rangarajan S., Hankey G.J., Oveisgharan S., Canavan M., McDermott C., Xavier D., Zhang H., Damasceno A. (2023). Alcohol Intake as a Risk Factor for Acute Stroke: The INTERSTROKE Study. Neurology.

[30] Berger K., Ajani U.A., Kase C.S., Gaziano J.M., Buring J.E., Glynn R.J., Hennekens C.H. (1999). Light-to-Moderate Alcohol Consumption and the Risk of Stroke Among U.S. Male Physicians. N. Engl. J. Med..

[31] Iso H., Baba S., Mannami T., Sasaki S., Okada K., Konishi M., Tsugane S., JPHC Study Group (2004). Alcohol Consumption and Risk of Stroke Among Middle-Aged Men: The JPHC Study Cohort I. Stroke.

[32] George M.G. (2020). Risk Factors for Ischemic Stroke in Younger Adults: A Focused Update. Stroke.

[33] Khan M., Wasay M., O’Donnell M.J., Iqbal R., Langhorne P., Rosengren A., Damasceno A., Oguz A., Lanas F., Pogosova N. (2023). Risk Factors for Stroke in the Young (18–45 Years): A Case-Control Analysis of INTERSTROKE Data from 32 Countries. Neuroepidemiology.

[34] Chuang Y.W., Yu T.M., Huang S.T., Sun K.T., Lo Y.C., Fu P.K., Lee B.J., Chen C.H., Lin C.L., Kao C.H. (2018). Young-Adult Polycystic Kidney Disease is Associated with Major Cardiovascular Complications. Int. J. Environ. Res. Public Health.

[35] Vlak M.H., Algra A., Brandenburg R., Rinkel G.J. (2011). Prevalence of Unruptured Intracranial Aneurysms, with Emphasis on Sex, Age, Comorbidity, Country, and Time Period: A Systematic Review and Meta-Analysis. Lancet Neurol..

[36] Chebib F.T., Tawk R.G. (2024). All Patients with ADPKD Should Undergo Screening for Intracranial Aneurysms: CON. Kidney360.

[37] Hung P.H., Lin C.H., Hung K.Y., Muo C.H., Chung M.C., Chang C.H., Chung C.J. (2020). Clinical Burden of Autosomal Dominant Polycystic Kidney Disease. Aging.

[38] Kidney Disease: Improving Global Outcomes (KDIGO) ADPKD Work Group (2025). KDIGO 2025 Clinical Practice Guideline for the Evaluation, Management, and Treatment of Autosomal Dominant Polycystic Kidney Disease (ADPKD). Kidney Int..

[39] Liao C.-H., Lin Y.-T., Tsai Y.-H., Chien H.-Y., Chen C.-C. (2025). The Impact of Autosomal Dominant Polycystic Kidney Disease on the Presence of Cerebral Microbleeds: A Case-Control Matched Study. J. Stroke Cerebrovasc. Dis..

[40] Kelleher C.L., McFann K.K., Johnson A.M., Schrier R.W. (2004). Characteristics of Hypertension in Young Adults with Autosomal Dominant Polycystic Kidney Disease Compared with the General U.S. Population. Am. J. Hypertens..

[41] Martínez V., Furlano M., Sans L., Pulido L., García R., Pérez-Gómez M.V., Sánchez-Rodríguez J., Blasco M., Castro-Alonso C., Fernández-Fresnedo G. (2022). Autosomal Dominant Polycystic Kidney Disease in Young Adults. Clin. Kidney J..

[42] Zhang Y., He D., Zhang W., Xing Y., Guo Y., Wang F., Jia J., Yan T., Liu Y., Lin S. (2020). ACE Inhibitor Benefit to Kidney and Cardiovascular Outcomes for Patients with Non-Dialysis Chronic Kidney Disease Stages 3–5: A Network Meta-Analysis of Randomised Clinical Trials. Drugs.

[43] Kataoka H., Akagawa H., Ushio Y., Sato M., Manabe S., Makabe S., Kawachi K., Akihisa T., Iwasa N., Yoshida R. (2022). Mutation Type and Intracranial Aneurysm Formation in Autosomal Dominant Polycystic Kidney Disease. Stroke Vasc. Interv. Neurol..

[44] Zuurbier C., Greving J.P., Rinkel G., Ruigrok Y.M. (2020). Higher Risk of Intracranial Aneurysms and Subarachnoid Haemorrhage in Siblings of Families with Intracranial Aneurysms. Eur. Stroke J..

[45] Mensing L.A., van Tuijl R.J., de Kort G.A., van der Schaaf I.C., Visseren F.L., Rinkel G.J., Velthuis B.K., Ruigrok Y.M., UCC-SMART Study Group (2023). Screening for Intracranial Aneurysms in Persons ≥35 Years with Hypertension and Atherosclerotic Disease Who Smoke(d). Eur. Stroke J..

[46] Catania M., De Rosa L.I., Kola K., Brambilla Pisoni M., Manunta P., Vezzoli G., Sciarrone Alibrandi M.T. (2024). Early Detection Matters: Bridging Evidence and Practice, a Call for Enhanced Cardiovascular Screening in ADPKD. Kidney Int. Rep..

[47] Boot E., Ekker M.S., Putaala J., Kittner S., De Leeuw F.-E., Tuladhar A.M. (2020). Ischaemic Stroke in Young Adults: A Global Perspective. J. Neurol. Neurosurg. Psychiatry.

[48] Stack C.A., Cole J.W., Dehkharghani S. (2021). Stroke in Young Adults. Stroke.

[49] Singhal A.B., Biller J., Elkind M.S., Fullerton H.J., Jauch E.C., Kittner S.J., Levine D.A., Levine S.R. (2013). Recognition and Management of Stroke in Young Adults and Adolescents. Neurology.

[50] Chen X., Zhao X., Xu F., Guo M., Yang Y., Zhong L., Weng X., Liu X. (2022). A Systematic Review and Meta-Analysis Comparing FAST and BEFAST in Acute Stroke Patients. Front. Neurol..

[51] Lin S.F., Chen C.I., Hu H.H., Bai C.-H. (2018). Predicting Functional Outcomes of Posterior Circulation Acute Ischemic Stroke in First 36 h of Stroke Onset. J. Neurol..

[52] Brouwer J., Smaal J.A., Emmer B.J., de Ridder I.R., van den Wijngaard I.R., de Leeuw F.E., Hofmeijer J., van Zwam W.H., Martens J.M., Roos Y.B.W.E.M. (2022). Endovascular Thrombectomy in Young Patients With Stroke: A MR CLEAN Registry Study. Stroke.

[53] Putaala J., Metso T.M., Metso A.J., Mäkelä E., Haapaniemi E., Salonen O., Kaste M., Tatlisumak T. (2009). Thrombolysis in Young Adults With Ischemic Stroke. Stroke.

[54] Dutta T., Ryan K.A., Thompson O., Lopez H., Fecteau N., Sparks M.J., Chaturvedi S., Cronin C., Mehndiratta P., Nunez Gonzalez J.R. (2021). Marijuana Use and the Risk of Early Ischemic Stroke: The Stroke Prevention in Young Adults Study. Stroke.

[55] Aycock D.M., Clark P.C., Hayat M.J., Salazar L.F., Eriksen M.P. (2023). Stroke Counseling Intervention for Young Adult African Americans: A Randomized Controlled Trial. Nurs. Res..

[56] Lo S.H.S., Chau J.P.C., Choi K.C., Shum E.W.C., Yeung J.H.M., Li S.H. (2021). Promoting Community Reintegration Using Narratives and Skills Building for Young Adults with Stroke: A Protocol for a Randomised Controlled Trial. BMC Neurol..

[57] Chan C.K.P., Lo T.L.T., Wan A.H.Y., Leung P.P.Y., Pang M.Y.C. (2021). A Randomised Controlled Trial of Expressive Arts-Based Intervention for Young Stroke Survivors. BMC Complement. Med. Ther..

[58] Li L., Scott C.A., Rothwell P.M. (2022). Association of Younger vs. Older Ages with Changes in Incidence of Stroke and Other Vascular Events, 2002–2018. JAMA.

[59] Huggins H.E., Brady M., Emma J.P., Thaler D.E., Leung L.Y. (2020). Differences in Presenting Symptoms of Acute Stroke among Young and Older Adults. J. Stroke Cerebrovasc. Dis..

[60] Fonarow G.C., Reeves M.J., Zhao X., Olson D.M., Smith E.E., Saver J.L., Schwamm L.H., Get With the Guidelines-Stroke Steering Committee and Investigators (2010). Age-Related Differences in Characteristics, Performance Measures, Treatment Trends, and Outcomes in Patients with Ischemic Stroke. Circulation.

[61] Lutski M., Zucker I., Shohat T., Tanne D. (2017). Characteristics and Outcomes of Young Patients with First-Ever Ischemic Stroke Compared to Older Patients: The National Acute Stroke ISraeli Registry. Front. Neurol..

[62] Patel U.K., Dave M., Lekshminarayanan A., Malik P., DeMasi M., Chandramohan S., Pillai S., Tirupathi R., Shah S., Jani V.B. (2021). Risk Factors and Incidence of Acute Ischemic Stroke: A Comparative Study Between Young Adults and Older Adults. Cureus.

[63] Matuja S.S., Munseri P., Khanbhai K. (2020). The Burden and Outcomes of Stroke in Young Adults at a Tertiary Hospital in Tanzania: A Comparison with Older Adults. BMC Neurol..

[64] Moosa A., Osama D., Alnidawi F., Algillidary S., Hussein A., Das P. (2023). Risk Factors, Incidence, and Outcome of Stroke: A Retrospective Cross-Sectional Hospital-Based Study Comparing Young Adults and Elderly. Cureus.

[65] Spence J.D. (2018). Diet for Stroke Prevention. Stroke Vasc. Neurol..

[66] Reddin C., Murphy R., Hankey G.J., Judge C., Xavier D., Rosengren A., Ferguson J., Alvarez-Iglesias A., Oveisgharan S., Iversen H.K. (2022). Association of Psychosocial Stress with Risk of Acute Stroke. JAMA Netw. Open.

[67] Sobalska A., Tomczyk K., Furman J., Łabuz-Roszak B. (2020). Assessment of Adult Eating Habits in the Nutritional Prevention of Stroke. Wiad. Lek..

[68] Mälstam E., Asaba E., Åkesson E., Guidetti S., Patomella A.-H. (2023). The Feasibility of Make My Day—A Randomized Controlled Pilot Trial of a Stroke Prevention Program in Primary Healthcare. Int. J. Environ. Res. Public Health.

[69] Von Sarnowski B., Putaala J., Grittner U., Gaertner B., Schminke U., Curtze S., Huber R., Tanislav C., Hölscher T., Engelhorn T. (2013). Lifestyle Risk Factors for Ischemic Stroke and Transient Ischemic Attack in Young Adults in the Stroke in Young Fabry Patients Study. Stroke.

[70] Loucks E.B., Schuman-Olivier Z., Britton W.B., Fresco D.M., Desbordes G., Brewer J.A., Fulwiler C. (2015). Mindfulness and Cardiovascular Disease Risk: State of the Evidence, Plausible Mechanisms, and Theoretical Framework. Curr. Cardiol. Rep..

[71] Calderone A., Latella D., Impellizzeri F., de Pasquale P., Famà F., Quartarone A., Calabrò R.S. (2024). Neurobiological Changes Induced by Mindfulness and Meditation: A Systematic Review. Biomedicines.

[72] Markidan J., Cole J.W., Cronin C.A., Merino J.G., Phipps M.S., Wozniak M.A., Kittner S.J. (2018). Smoking and Risk of Ischemic Stroke in Young Men. Stroke.

[73] Fisher M., Lees K., Spence J.D. (2006). Nutrition and Stroke Prevention. Stroke.

[74] Amoah D., Schmidt M., Mather C., Prior S., Herath M.P., Bird M.-L. (2024). An International Perspective on Young Stroke Incidence and Risk Factors: A Scoping Review. BMC Public Health.

[75] Dearborn J.L., Urrutia V.C., Kernan W.N. (2015). The Case for Diet: A Safe and Efficacious Strategy for Secondary Stroke Prevention. Front. Neurol..

[76] Apostolopoulou M., Michalakis K., Miras A., Hatzitolios A., Savopoulos C. (2012). Nutrition in the Primary and Secondary Prevention of Stroke. Maturitas.

[77] Si Larbi M., Lahmidi S., Boukari A.K., Boukeloua A. (2021). Ischemic and Non-ischemic Stroke in Young Adults—A Look at Risk Factors and Outcome in a Developing Country. Cureus.

[78] Martens L.G., Luo J., Willems van Dijk K., Jukema J.W., Noordam R., van Heemst D. (2021). Diet-Derived Antioxidants Do Not Decrease Risk of Ischemic Stroke: A Mendelian Randomization Study in 1 Million People. J. Am. Heart Assoc..

[79] Ascherio A., Rimm E.B., Hernán M.A., Giovannucci E., Kawachi I., Stampfer M.J., Willett W.C. (1999). Relation of Consumption of Vitamin E, Vitamin C, and Carotenoids to Risk for Stroke Among Men in the United States. Ann. Intern. Med..

[80] Boccardi V., Tagliafico L., Persia A., Page E., Ottaviani S., Cremonini A.L., Borgarelli C., Pisciotta L., Mecocci P., Nencioni A. (2024). The Potential Effects of Red Wine and Its Components on Neurocognitive Disorders: A Narrative Review. Nutrients.

[81] Strazzullo P., D’Elia L., Kandala N.B., Cappuccio F.P. (2009). Salt Intake, Stroke, and Cardiovascular Disease: Meta-Analysis of Prospective Studies. BMJ.

[82] Sarikaya H., Ferro J., Arnold M. (2015). Stroke Prevention—Medical and Lifestyle Measures. Eur. Neurol..

[83] Prior P.L., Suskin N. (2018). Exercise for Stroke Prevention. Stroke Vasc. Neurol..

[84] Lee C.D., Folsom A.R., Blair S.N. (2003). Physical Activity and Stroke Risk: A Meta-Analysis. Stroke.

[85] Howard V.J., McDonnell N.M. (2015). Physical Activity in Primary Stroke Prevention: Just Do It!. Stroke.

[86] Viktorisson A., Palstam A., Nyberg F., Berg C., Lissner L., Sunnerhagen K.S. (2024). Domain-Specific Physical Activity and Stroke in Sweden. JAMA Netw. Open.

[87] De Santis F., Romoli M., Foschi M., Sciancalepore F.D., D’Anna L., Barba L., Abu-Rumeileh S., Sacco S., Ornello R. (2024). Risk of Stroke with Different Levels of Leisure-Time Physical Activity: A Systematic Review and Meta-Analysis of Prospective Cohort Studies. J. Neurol. Neurosurg. Psychiatry.

[88] Grau A.J., Barth C., Geletneky B., Ling P., Palm F., Lichy C., Becher H., Buggle F. (2009). Association between Recent Sports Activity, Sports Activity in Young Adulthood, and Stroke. Stroke.

[89] Reinholdsson M., Palstam A., Sunnerhagen K.S. (2018). Prestroke Physical Activity Could Influence Acute Stroke Severity (Part of PAPSIGOT). Neurology.

[90] Ohlsson C., Bygdell M., Sondén A., Jern C., Rosengren A., Kindblom J.M. (2017). BMI Increase through Puberty and Adolescence Is Associated with Risk of Adult Stroke. Neurology.

[91] Booth J., Connelly L., Lawrence M., Chalmers C., Joice S., Becker C., Dougall N. (2015). Evidence of Perceived Psychosocial Stress as a Risk Factor for Stroke in Adults: A Meta-Analysis. BMC Neurol..

[92] Wake A., O’Donnell A.W. (2024). Longitudinal Relationships Between Financial Stress, Career-Related Optimism, and Psychological Distress During Emerging Adulthood in Australia. Youth Soc..

[93] Kim B.J., Cho I.S., Cho K.I. (2017). Impact of Mindfulness-Based Stress Reduction Therapy on Myocardial Function and Endothelial Dysfunction in Female Patients with Microvascular Angina. J. Cardiovasc. Ultrasound.

[94] Liu D., Yang L., Liu P., Wang Y., Gao L. (2024). Impact of Cannabis Abuse on the Occurrence of Stroke in Young People: A Systematic Review and Meta-Analysis. Front. Neurol..

[95] Centers for Disease Control and Prevention (CDC) Smoking and Cardiovascular Disease. https://www.cdc.gov/tobacco/about/cigarettes-and-cardiovascular-disease.html.

[96] Shinton R., Beevers G. (1989). Meta-Analysis of Relation Between Cigarette Smoking and Stroke. BMJ.

[97] Noubiap J.J., Fitzgerald J.L., Gallagher C., Thomas G., Middeldorp M.E., Sanders P. (2021). Rates, Predictors, and Impact of Smoking Cessation after Stroke or Transient Ischemic Attack: A Systematic Review and Meta-Analysis. J. Stroke Cerebrovasc. Dis..

[98] Wechsler P.M., Liberman A.L., Restifo D., Abramson E.L., Navi B.B., Kamel H., Parikh N.S. (2023). Cost-Effectiveness of Smoking Cessation Interventions in Patients with Ischemic Stroke and Transient Ischemic Attack. Stroke.

[99] Lambiase M.J., Kubzansky L.D., Thurston R.C. (2015). Positive Psychological Health and Stroke Risk: The Benefits of Emotional Vitality. Health Psychol..

[100] Park C.S., Choi E.K., Han K.D., Ahn H.J., Kwon S., Lee S.R., Oh S., Lip G.Y.H. (2023). Increased cardiovascular events in young patients with mental disorders: A nationwide cohort study. Eur. J. Prev. Cardiol..

[101] Ramasubbu R. (2011). Therapy for prevention of post-stroke depression. Expert Opin. Pharmacother..

[102] Greenlund K.J., Giles W.H., Keenan N.L., Croft J.B., Mensah G.A. (2002). Physician Advice, Patient Actions, and Health-Related Quality of Life in Secondary Prevention of Stroke through Diet and Exercise. Stroke.

[103] Rizk H.I., Magdy R., Emam K., Mohammed M.S., Aboulfotooh A.M. (2024). Substance use disorder in young adults with stroke: Clinical characteristics and outcome. Acta Neurol. Belg..

[104] George M.G., Matters M.D., McGruder H.F., Valderrama A.L., Xie J. (2008). The Role of Public Health in Promoting Quality Improvement in Care for Stroke and Heart Disease. Prev. Chronic Dis..

[105] Centers for Disease Control and Prevention Preventing Stroke: Tips for Prevention. MMWR Morb Mortal Wkly Rep..

[106] Lambert C., Chang W., Parker R., Allen K., Stevens L., Blood J., Nystrom K., Forman R. (2024). Enhancing Stroke Knowledge Among Youth: Insights from Stroke Busters. J. Stroke Cerebrovasc. Dis..

[107] Pratt C.A., Ha L., Levine S.R., Pratt C.B. (2003). Stroke Knowledge and Barriers to Stroke Prevention among African Americans: Implications for Health Communication. J. Health Commun..

[108] Iskandar K., Rahme D., Salameh P., Haddad C., Sacre H., Bahlol M., Darwish R.M., El Khatib S., Safwan J., Sakr F. (2024). Evaluating the Influence of a 3-Min Online Video on the Community Knowledge of Stroke in Four Arab Countries. Front. Public Health.

[109] Hartigan I., O’Connell E., O’Brien S., Weathers E., Cornally N., Kilonzo B., McCarthy G. (2014). The Irish National Stroke Awareness Campaign: A Stroke of Success?. Appl. Nurs. Res..

[110] Kleindorfer D.O., Towfighi A., Chaturvedi S., Cockroft K.M., Gutierrez J., Lombardi-Hill D., Kamel H., Kernan W.N., Leira E.C., Maas M.B. (2021). 2021 Guideline for the Prevention of Stroke in Patients with Stroke and Transient Ischemic Attack: A Guideline from the American Heart Association/American Stroke Association. Stroke.

